# Seasonal Succession and Spatial Patterns of *Synechococcus* Microdiversity in a Salt Marsh Estuary Revealed through 16S rRNA Gene Oligotyping

**DOI:** 10.3389/fmicb.2017.01496

**Published:** 2017-08-09

**Authors:** Katherine R. M. Mackey, Kristen Hunter-Cevera, Gregory L. Britten, Leslie G. Murphy, Mitchell L. Sogin, Julie A. Huber

**Affiliations:** ^1^Earth System Science, University of California Irvine Irvine, CA, United States; ^2^Marine Biological Laboratory, Josephine Bay Paul Center for Comparative Molecular Biology and Evolution Woods Hole, MA, United States

**Keywords:** oligotyping, *Synechococcus*, salt marsh estuary, succession, temperature, salinity

## Abstract

*Synechococcus* are ubiquitous and cosmopolitan cyanobacteria that play important roles in global productivity and biogeochemical cycles. This study investigated the fine scale microdiversity, seasonal patterns, and spatial distributions of *Synechococcus* in estuarine waters of Little Sippewissett salt marsh (LSM) on Cape Cod, MA. The proportion of *Synechococcus* reads was higher in the summer than winter, and higher in coastal waters than within the estuary. Variations in the V4–V6 region of the bacterial 16S rRNA gene revealed 12 unique *Synechococcus* oligotypes. Two distinct communities emerged in early and late summer, each comprising a different set of statistically co-occurring *Synechococcus* oligotypes from different clades. The early summer community included clades I and IV, which correlated with lower temperature and higher dissolved oxygen levels. The late summer community included clades CB5, I, IV, and VI, which correlated with higher temperatures and higher salinity levels. Four rare oligotypes occurred in the late summer community, and their relative abundances more strongly correlated with high salinity than did other co-occurring oligotypes. The analysis revealed that multiple, closely related oligotypes comprised certain abundant clades (e.g., clade 1 in the early summer and clade CB5 in the late summer), but the correlations between these oligotypes varied from pair to pair, suggesting they had slightly different niches despite being closely related at the clade level. Lack of tidal water exchange between sampling stations gave rise to a unique oligotype not abundant at other locations in the estuary, suggesting physical isolation plays a role in generating additional microdiversity within the community. Together, these results contribute to our understanding of the environmental and ecological factors that influence patterns of *Synechococcus* microbial community composition over space and time in salt marsh estuarine waters.

## Introduction

The genus *Synechococcus* is a remarkably diverse group that inhabits aquatic environments ranging from open ocean and coastal waters (Bouman et al., 2006; Zwirglmaier et al., 2007, 2008; Post et al., 2011; Flombaum et al., 2013; Sohm et al., 2015; Hunter-Cevera et al., 2016) to brackish estuaries (Chen et al., 2006; Choi and Noh, 2009), and freshwater lakes (Ivanikova et al., 2008). Despite occupying this extensive range of environments, little is known about the patterns of abundance, diversity, and distribution of *Synechococcus* in estuary waters relative to coastal waters and the open ocean. Estuaries are dynamic environments where freshwater and seawater mix, generating hydrographic gradients that vary spatially and over tidal and seasonal cycles. These habitats therefore have distinctive gradients, making them attractive sites to investigate *Synechococcus* community dynamics.

The diversity of *Synechococcus* based on 16S rRNA gene phylogeny spans three sub-clusters (5.1, 5.2, and 5.3), and further resolution of the genus is possible using additional genetic markers such as the internally transcribed spacer (ITS) region and the genes *cpeA, narB, ntcA, petB, rbcL*, and *rpoC1* (Scanlan et al., 2009; Ahlgren and Rocap, 2012). These markers partition the genus into at least 20 distinct clades (Scanlan et al., 2009; Ahlgren and Rocap, 2012; Huang et al., 2012; Mazard et al., 2012; Sohm et al., 2015). The majority of marine strains fall within sub-cluster 5.1. Efforts to link clade identity to ecological or physiological characteristics suggest that *Synechococcus* has diversified based on temperature (Mackey et al., 2013; Pittera et al., 2014), nutrient type and availability (Glover et al., 1988; Moore et al., 2002; Saito et al., 2005; Palenik et al., 2006; Mackey et al., 2015; Sohm et al., 2015), and light intensity and spectrum (Palenik, 2001; Everroad et al., 2006; Mackey et al., 2017). For example, clade III is found in warm, oligotrophic waters, while clades I and IV tend to dominate colder, nutrient rich waters, and clade II is more broadly distributed throughout the tropics and subtropics (Mazard et al., 2012; Sohm et al., 2015). However, niches occupied by *Synechococcus* strains are generally more flexible and have more overlap compared to ecotypes of the closely related genus *Prochlorococcus*. This may be due to extensive lateral gene transfer or independent parallel evolution of traits that occurred after the marine *Synechococcus* lineages diverged (Ahlgren and Rocap, 2012; Sohm et al., 2015).

A common observation within *Synechococcus* populations is the co-occurrence of different clades. Co-occurrence patterns appear to be particularly dynamic in space and time in estuaries. A comparison of *Synechococcus* populations from estuarine and coastal waters in the Pearl River Estuary in Hong Kong showed that clades II and IX dominated both sites in the winter, whereas in summer the coastal population comprised clades II and VI, and the estuarine population comprised co-occurring marine and freshwater taxa (Xia et al., 2015). This shift was attributed to warmer temperatures and freshwater input during the summer monsoon. In contrast, freshwater taxa were rare in the Chesapeake Bay estuary even at low salinity sites, and populations were instead dominated by co-occurring members of clades CB4 an CB5 (Chen et al., 2006). Coastal waters also show co-occurrence patterns. In the California Current, clades II and III co-occurred in the period leading up to the spring bloom, and clades I and IV dominated during the bloom itself (Tai and Palenik, 2009). In the Red Sea, co-existence of clades V, VI, and X was observed during transitional periods between mixing and stratification (Post et al., 2011). Very little is known about the factors that support coexistence among these closely related organisms. The greater genetic diversity of the aggregate *Synechococcus* population may effectively expand the overall niche filled by this genus by out-competing other genera. In some cases, less abundant *Synechococcus* clades may persist at low, but stable, levels by avoiding top down pressures like grazing and viral infection, both of which are more impactful when cell numbers are higher (Pedrós-Alió, 2006; Sogin et al., 2006; Fuhrman, 2009). Low-abundance strains can influence biogeochemical processes (Gilbert et al., 2012) and may serve as important reservoirs of genetic diversity (Sogin et al., 2006). The mechanisms that give rise to coexistence of high- and low-abundance strains is not well-understood, although their co-occurrence patterns are routinely observed (Ahlgren and Rocap, 2012; Sohm et al., 2015). Methods that examine small-scale diversity among co-occurring strains can provide a window into the factors that influence dynamics of rare as well as closely related but distinct populations.

To explore the patterns of estuarine *Synechococcus* diversity in space and time, we used an oligotyping approach to examine populations in estuarine waters of the Little Sippewissett salt marsh located in Cape Cod, MA. Oligotyping is a method that differentiates between closely related members of a microbial community (Eren et al., 2013) and enables fine scale diversity within microbial communities to be identified. The technique uses entropy analysis of variable sites in related sequences identified via taxonomic classification or clustering of high throughput sequencing data (Nicholson et al., 1987; Eren et al., 2013; Wilbanks et al., 2014). We hypothesized that oligotypes would report seasonal community dynamics and co-occurrence patterns as has been done in previous studies at the clade (Chen et al., 2006; Choi and Noh, 2009) and subclade (Tai and Palenik, 2009; Paerl et al., 2011; Robidart et al., 2012) levels. We identified 12 *Synechococcus* oligotypes, show that their relative abundances vary seasonally and spatially within the estuary, and explore their patterns of co-occurrence and succession in light of their fine-scale diversity.

## Materials and methods

### Site description and sample collection

**Figure 1:**
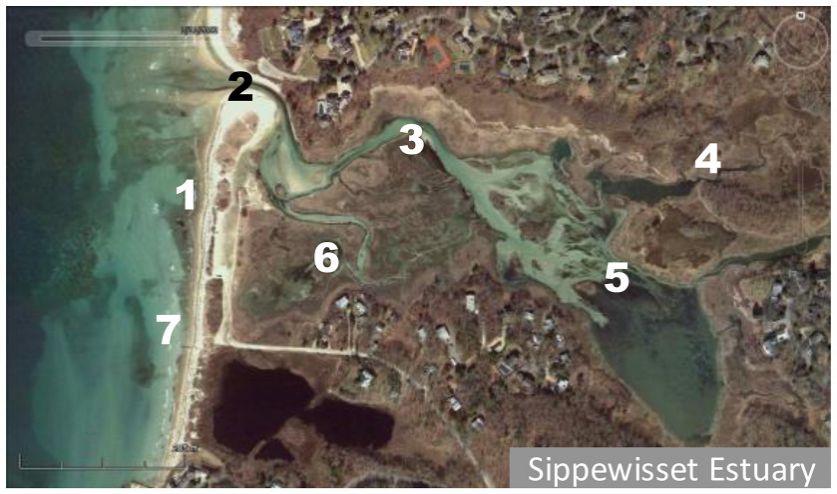
Little Sippewissett salt marsh estuary sampling stations.

The estuary of Little Sippewissett salt marsh is located on the southwestern tip of Cape Cod in the town of Falmouth, MA (41.576°N, 70.636°W). The estuary empties into Buzzards Bay on the western side of the Cape. Samples were collected and processed as described in Eren et al. (2013). Briefly, surface water was collected during low tide from seven stations within the marsh and nearby coastal waters (Figure 1). Two stations were in coastal waters (1 and 7), four were within the main channel of the estuary (2, 3, 4, and 5) and one was in a spatially segregated shallow site within the marsh (station 6). Depending on the tidal cycle and amplitude, station 6 at times only had several centimeters of water depth. Sampling was done weekly from May 31, 2007 until September 4, 2007, and then monthly until September 2008.

One-liter water samples were stored on ice until filtration onto 0.22 μm pore size Sterivex filter cartridges. Dissolved oxygen and temperature were measured for each sample with a hand-held probe (Yellow Spring Instrument, YSI), and daily precipitation values were obtained from the Global Historical Climatology Network (GHCN) online database (https://climexp.knmi.nl/) of rain gauge measurements in Vineyard Haven, MA (41.39°N, 70.61°W) ~8 miles offshore of Sippewissett Marsh. DNA was extracted and purified using a modified salt precipitation method as described previously (Sinigalliano et al., 2007; Eren et al., 2013). Bacterial 16S rRNA gene amplicons spanning the V4 through V6 regions were amplified using fusion primers, sequenced from the V6 end on a Roche GS-FLX 454 instrument using Titanium protocols, and quality-filtered and trimmed as described previously (Filkins et al., 2012). Raw sequences are deposited in the NCBI Sequence Read Archive (SRP017571: PRJNA183456).

### Oligotyping

Within-genus diversity of *Synechococcus* was resolved using the oligotyping procedure as described previously (Eren et al., 2013), following recommended procedures described at http://merenlab.org/2013/11/04/oligotyping-best-practices/. Oligotyping is a computational method that identifies fine-scale diversity based on subtle variations in 16S RNA gene sequences. Oligotyping improves the resolution with which diversity can be identified because oligotype identification is not based on the availability of existing sequences within reference databases (as in taxonomic classification), and resolution of fine-scale diversity is not restricted by the choice of similarity threshold value (as in cluster analysis). The technique uncovers microdiversity within a given taxon or operational taxonomic unit (OTU), and is particularly useful when applied to taxonomic assignments or OTUs that occur throughout a dataset and may respond to changing environmental conditions (Eren et al., 2013).

Sequences identified as *Synechococcus* based on GAST taxonomic assignments were aligned with PyNAST aligner (version 0.1) against a GreenGenes OTU alignment template (version 6 Oct2010). Of the 13,427 *Synechococcus* sequences, only one failed to align. Mean length of *Synechococcus* reads was 482 base pairs with a standard deviation of 1.74 base pairs. Uninformative gap regions were removed and the entropy of each nucleotide position was calculated within the oligotype package. Nucleotide positions used to define oligotypes were selected from an iterative process; positions were included if they contributed to converged entropy within an oligotype and excluded if they contained redundant information of another position or did not contribute to convergence. A total of 19 positions were used to define oligotypes, and each oligotype was required to have a minimum substantive abundance of 20 (“M” parameter), such that an oligotype was not included if the most common sequence for that type occurred <20 times. Oligotypes were not required to comprise a certain percentage of reads, represent a minimum number of reads in a sample, or appear in more than one sample. Twelve oligotypes were identified and represented ~80% of the total *Synechococcus* reads. Reads that were not identified as belonging to an oligotype are collectively referred to as “other *Synechococcus*” in the discussion below.

The sequence read counts for oligotypes presented here are normalized to either total microbial read counts or total *Synechococcus* read counts. As such, these data show changes in relative abundance, and do not necessarily reflect actual changes in cell concentrations of a given oligotype. For example, an increase in the relative abundance of a given oligotype could be a result of a true increase in the cell concentration of that oligotype, but could also result if the concentration of another oligotype decreases. Therefore, the data presented here indicates whether an oligotype becomes a larger or smaller fraction of the community, but do not necessarily indicate increases or decreases in absolute cell abundance.

### Clade identification

For each representative oligotype sequence, we inferred a clade designation by matching the representative V4–V6 sequences for each oligotype to a reference database of 16S rRNA gene sequences from cultured *Synechococcus*. The database consisted of sequences for which unambiguous clade assignments have been determined with a higher resolution diversity marker (i.e., ITS, *petB, rpoC1, ntcA*). All sequences were downloaded from Genbank and clade classifications were obtained from the following sources (Fuller et al., 2003; Choi and Noh, 2009; Mazard et al., 2011, 2012; Ahlgren and Rocap, 2012; Hunter-Cevera et al., 2016). Representative V4–V6 sequences for each oligotype and database sequences were aligned with Clustal W (embedded within BioEdit, version 7.2.0, Hall 1999), and exact matches between oligotype and database sequences were identified. When no direct match was found, the closest sequence was used to infer a clade designation.

### Statistical analyses

To statistically group oligotypes according to their co-occurrence, we computed the principal components (PCs) with respect to a sample matrix of *Synechococcus* oligotype reads normalized to total *Synechococcus* reads for that sample. We then projected each oligotype sample vector onto the first two PCs of the matrix (Anderson, 2003). The relative strength of oligotype groupings was then quantified as the Euclidean distance of each group's centroid to the centroid of other groups in the space of the first two PCs. To investigate environmental correlates of oligotype grouping, we computed the multiple regression of each of the first two PCs against the following environmental variables: (i) water temperature, (ii) salinity, (iii) dissolved oxygen and (iv) weekly precipitation. The most important set of environmental correlates for each PC was defined as the set that minimizes the Bayesian Information Criterion (BIC; Kass and Raftery, 1995) for all possible multiple regressions with respect to the four environmental variables.

## Results

### *Synechococcus* oligotype patterns

**Figure 2:**
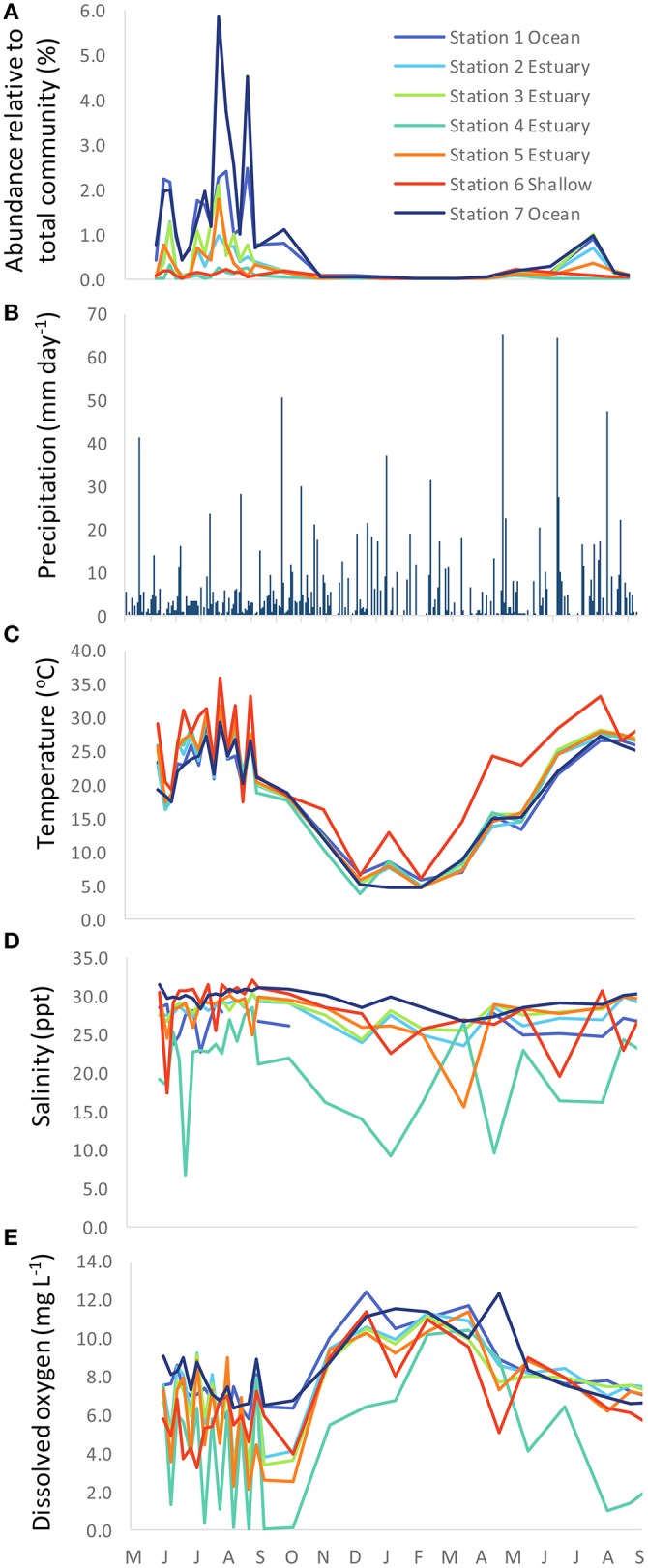
Time series of **(A)** relative abundance of *Synechococcus* (as *Synechococcus* reads relative to the total number of microbial reads for each sample), **(B)** precipitation, **(C)** temperature, **(D)** salinity, and **(E)** dissolved oxygen.

A total of 3,142,957 PCR amplicon sequence reads were generated from microbial populations of 189 samples collected at seven stations (Figure 1) on 27 different dates throughout 2007 and 2008. Of these sequences, 13,358 reads were classified as *Synechococcus*. The number of *Synechococcus* reads relative to the total microbial population varied spatially and seasonally, ranging from 0% on some winter sampling dates/locations up to 5.9% at coastal station 7 during the summer (Figure 2).

**Figure 3:**
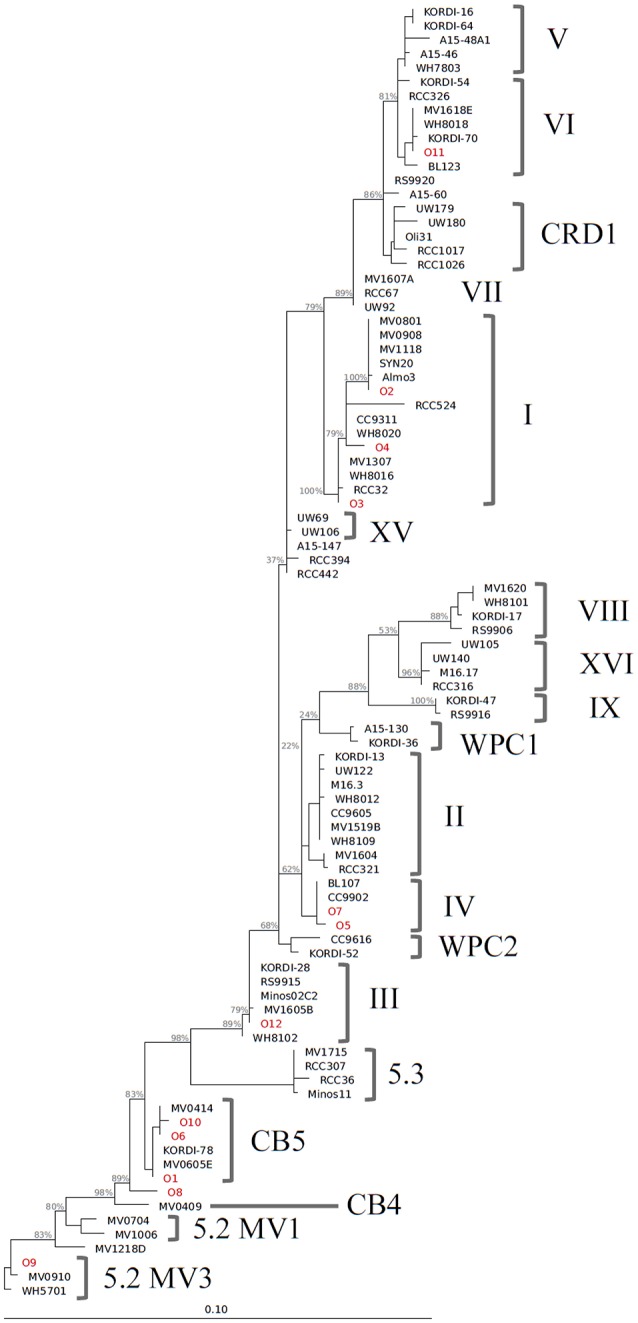
Phylogenetic tree constructed from 16S rRNA sequences (~1,200 bp) of *Synechococcus* clade representatives and representative V4–V6 sequences for each oligotype. Reconstruction was performed with the ARB software package (version 5.3, Ludwig et al., 2004) with a maximum likelihood approach using RAxML and a GTR GAMMA rate substitution model. Due to their shorter sequence length, oligotype sequences (indicated in red) were added to this base tree using the ARB parsimony method. Bootstrap analysis for support of tree branches was also carried out in ARB with rapid bootstrap analysis and 500 sample trees. Values for major branches appear in light gray.

**Figure 4:**
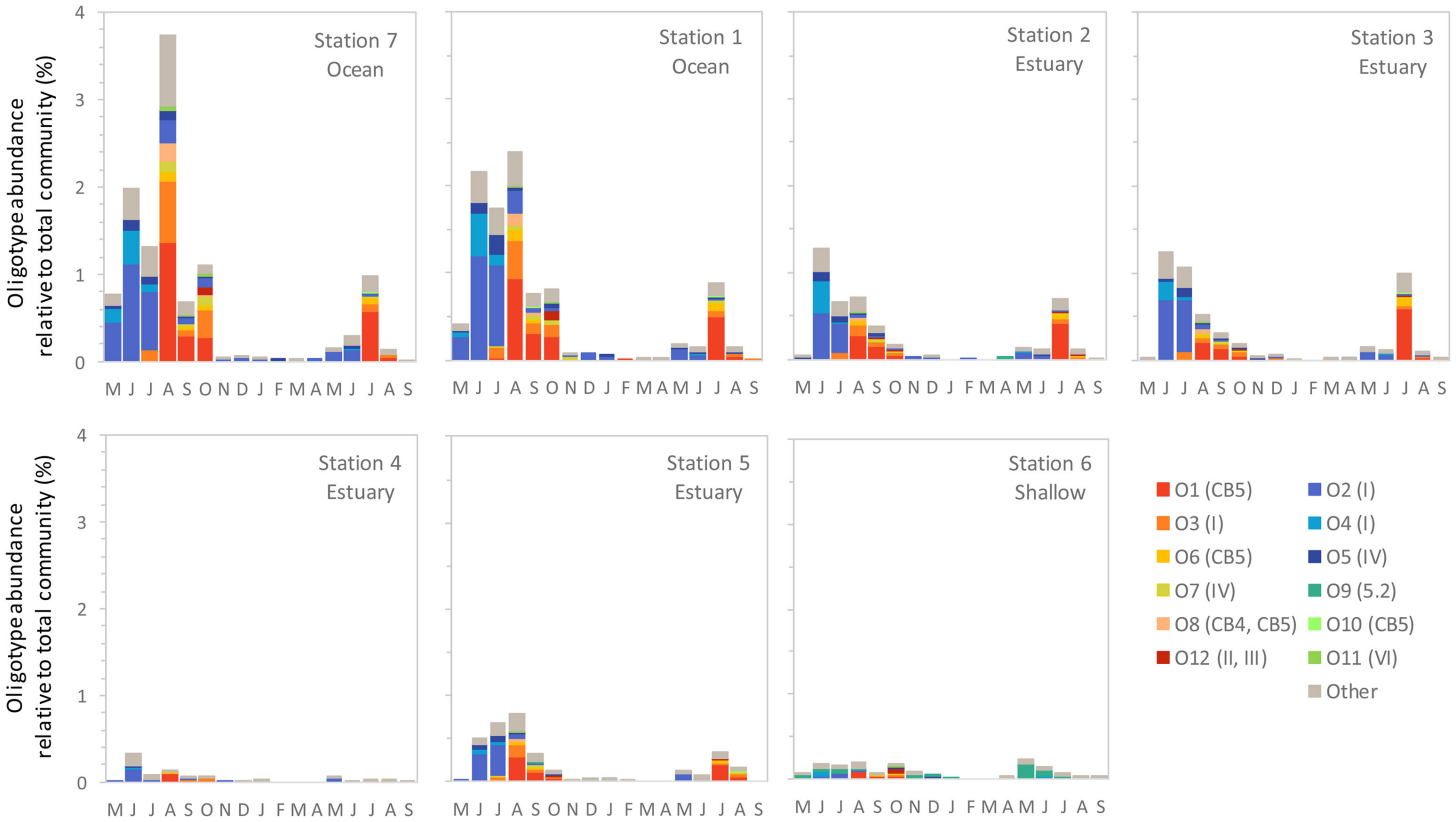
The monthly relative abundance of *Synechococcus* oligotypes (relative to total microbial reads for each sample) from May 2007 through September 2008. Legend shows oligotype number with clade identification in parentheses.

We identified 12 *Synechococcus* oligotypes, which together accounted for an average of 73% of the *Synechococcus* reads over the entire dataset and comprised 0–100% of the total *Synechococcus* population depending on sampling location and date. These oligotypes were affiliated with several different *Synechococcus* clades based on sequence similarity of the representative V4–V6 sequence for each oligotype to known cultured isolates, and each of the 12 representative V4–V6 sequence for each oligotype mapped perfectly or within several base pairs to a range of known *Synechococcus* clades (Figure 3; Table 1). The 12 oligotypes shared >95% V4-V6 sequence similarity (Table 2). Figure 3 shows the placement of these 12 oligotypes within the known *Synechococcus* phylogeny. The remaining reads not assigned to an oligotype were enumerated as “other *Synechococcus*” in our analysis. Clear seasonal and spatial patterns were apparent; *Synechococcus* comprised a greater fraction of the microbial population in the summer than in the winter, and had higher relative abundances in the coastal ocean compared to stations within the estuary (Figure 4).

**Table 1:** Clade affiliations of the 12 V4–V6 representative sequences for each *Synechococcus* oligotype.

**Oligotype**	**Clade best match**	**Number of base pairs different from best match**
O1	CB5	0 (exact match)
O2	I	0 (exact match)
O3	I	0 (exact match)
O4	I	2
O5	IV	1
O6	CB5	0 (exact match)
O7	IV	0 (exact match)
O8	CB4, CB5	7
O9	5.2	0 (exact match)
O10	CB5	1
O11	VI	0 (exact match)
O12	II, III	0 (exact match)

**Table 2:** Percent sequence similarity between V4 and V6 representative sequences for each oligotype.

	**O1**	**O2**	**O3**	**O4**	**O5**	**O6**	**O7**	**O8**	**O9**	**O10**	**O11**	**O12**
O1	–	96.36	96.15	95.75	97.17	99.80	97.37	98.58	95.55	99.60	96.96	97.17
O2		–	99.19	99.19	97.98	96.15	98.18	96.96	94.74	95.95	97.57	98.58
O3			–	99.39	98.79	96.36	98.58	96.56	94.94	96.15	98.18	98.99
O4				–	98.18	95.95	97.98	96.56	94.74	95.75	97.98	98.38
O5					–	97.37	99.80	96.36	94.53	97.17	98.58	99.39
O6						–	97.17	98.38	95.75	99.80	97.17	96.96
O7							–	96.56	94.33	96.96	98.38	99.60
O8								–	95.95	98.18	96.56	96.76
O9									–	95.95	94.53	94.33
O10										–	96.96	96.76
O11											–	98.79
O12												–

**Figure 5:**
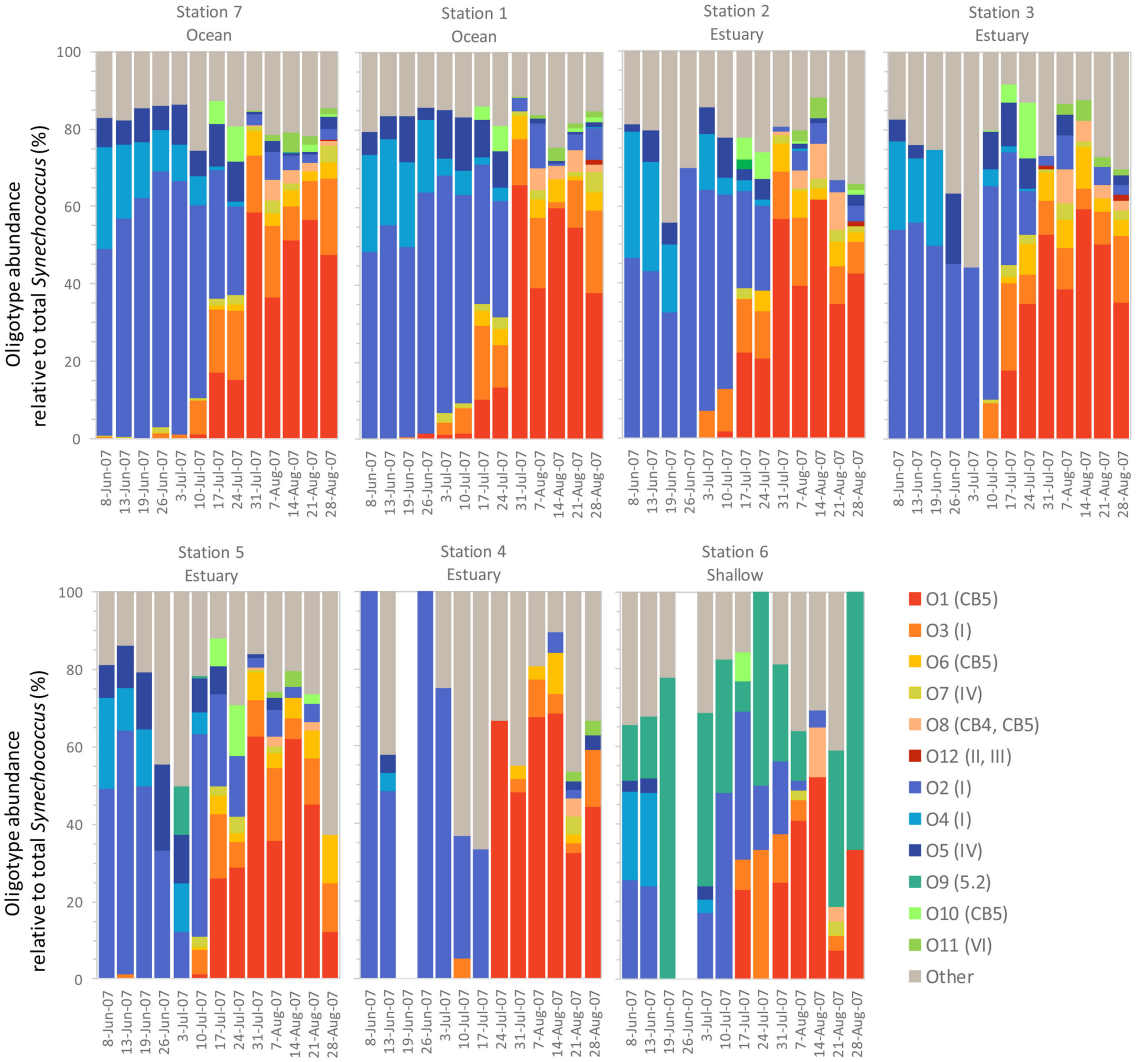
Summer weekly relative abundance of *Synechococcus* oligotypes (oligotype reads normalized to total *Synechococcus* reads for each sample). The summer time series shows two distinct communities, one in the early summer and one in the late summer, each dominated by a different set of co-occurring *Synechococcus* oligotypes. Blank columns in Station 4 and 6 indicate no *Synechococcus* reads were detected on those dates. Legend shows oligotype number with clade identification in parentheses.

Within the weekly summer samples, succession of oligotypes occurred between early and late summer (Figure 5). In the early summer beginning in June, oligotype O2 dominated the *Synechococcus* community. Oligotypes O4 and O5 also reached maximum values early in the summer, although at relative abundances below oligotype O2. Toward the end of the summer the *Synechococcus* community consisted of oligotypes O1, O3, O6, O7, O8, O10, and O11. The seasonal succession was apparent at each station, though oligotype 9 was only strongly represented in the shallow station 6 where it comprised a larger segment of the overall *Synechococcus* community (up to 100% of *Synechococcus* reads) than at other sites. Oligotypes 6, 8, 10, 11, and 12 were rare overall, and their relative abundances were not closely linked to any one station throughout the year (Figure S1).

Different patterns were observed between co-occurring oligotypes in the early and late summer communities. Oligotypes that comprised the majority of the early summer community tended to be well-correlated with each other (Figure S2). Relative abundance of these types showed consistent ratios: the relative abundance of O2 (clade I) was usually two-fold >O4 (clade I) and four-fold >O5 (clade IV). In contrast, correlations among oligotypes that comprised the late summer community were more variable. Strong correlations were observed among the most abundant oligotypes (O1 and O3, R2 = 0.87, O1 and 06, R2 = 0.96 and O3 and O6 = 0.83). Lower abundant oligotypes did not show strong correlations, despite belonging to the same clades (i.e., CB5).

### Environmental variables

Salinity values along the main channel of the estuary remained above 15 psu with the exception of station 4, the location furthest upstream toward the freshwater end member (Figure 2D). Station 4 also showed the greatest seasonal variation in salinity, with fresher waters occurring during the winter months. Temperature ranged from ~4°C to >35°C seasonally, with the highest temperatures observed in the shallow station 6 during summer months (Figure 2C). Dissolved oxygen varied among stations, with the highest concentrations observed in the coastal stations 1 and 7, and the lowest values observed in the freshwater end member station 4 (Figure 2E).

**Figure 6:**
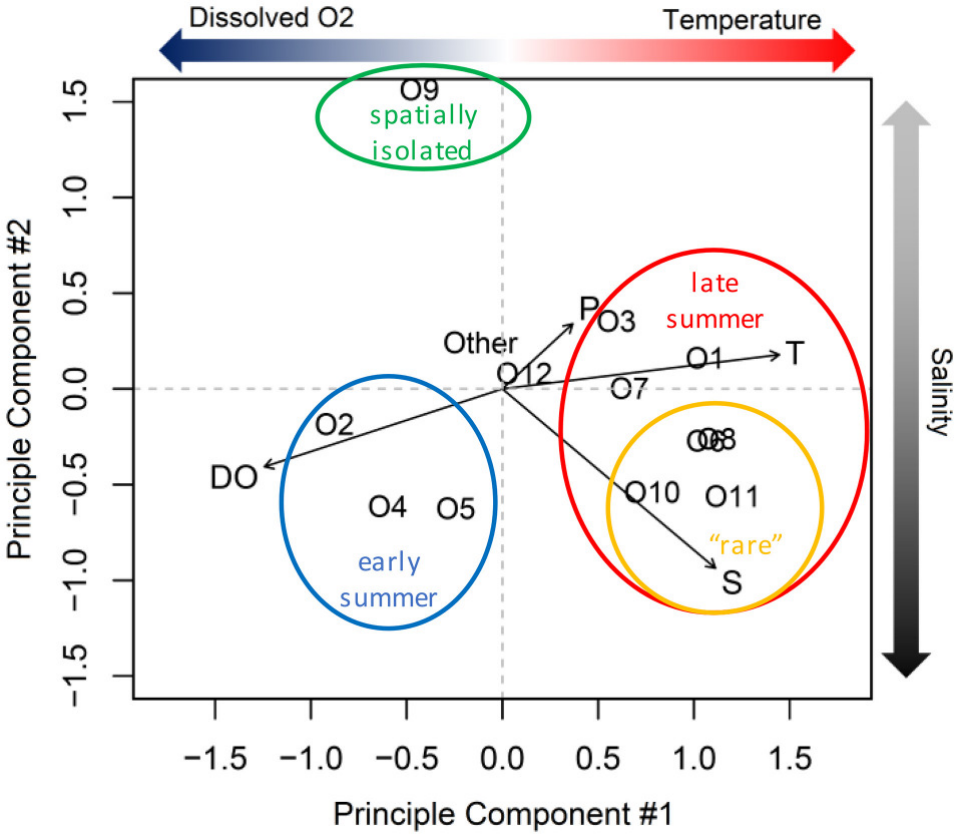
Principle component analysis of *Synechococcus* oligotype relative abundance. T, temperature; P, precipitation; S, salinity; DO, dissolved oxygen.

From principle component analysis (PCA) of oligotype relative abundances, we found that physical and chemical conditions in the estuary correlated with the patterns of oligotype co-occurrence (Figure 6). The first principle component captured 46% of the variance in oligotype relative abundance and discriminated oligotypes according to dissolved oxygen and temperature based on the multiple regression model selection using BIC. The second principle component explained an additional 21% of the variation and discriminated oligotypes according to salinity alone.

The statistical relationships among the dominant PCs and the environmental variables were consistent with the composition shift observed during the summer (Figure 5). Specifically, oligotypes 2, 4, and 5 correlated with lower temperatures and higher dissolved oxygen content based on the PCA. The shift in late summer to oligotypes 1, 3, and 7 correlated with higher temperatures. A third group of co-occurring late summer oligotypes (the comparatively lower relative abundance “rare” oligotypes 6, 8, 10, and 11) correlated with both PC1 and PC2. Oligotype 12 was not affiliated with either principle component or site, but became more abundant in the fall after the dominance of oligotypes 1, 3, and 7 in late summer had begun to dissipate. Oligotype 9 was also not strongly correlated with PC1 or PC1.

## Discussion

### Temporal diversity patterns

*Synechococcus* oligotypes comprised a larger proportion of the microbial community in the spring and summer months of June through September compared to winter months (Figure 4). Two distinct *Synechococcus* communities emerged that dominated in the early summer (May/June) and late summer (July onward), each composed of different, co-occurring *Synechococcus* oligotypes (Figure 5).

The *Synechococcus* community in early summer consisted of clade I oligotypes O2 and O4, and clade IV oligotype O5. Clades I and IV commonly co-occur in coastal waters, such as the cool waters off the coast of California (Tai and Palenik, 2009), as well as the open ocean at higher latitudes (Zwirglmaier et al., 2007, 2008). Tai and Palenik observed that while clade IV was typically more abundant than clade I in the open ocean, the relative dominance of the two clades fluctuated along the California coast, with clade I dominating for part of the bloom cycle (Tai and Palenik, 2009). In contrast, in this study, clade I outnumbered clade IV throughout the summer months. Similar patterns were observed at the nearby coastal water Martha's Vineyard Coastal Observatory (MVCO), located southeast of Sippewissett Marsh, where the spring bloom of *Synechococcus* was mainly comprised of clade I representatives (Hunter-Cevera et al., 2016), and is correlated with warming water temperatures in the spring.

In contrast to the early summer community, the late summer community correlated with higher temperature and, for some oligotypes, higher salinity levels (Figure 6). This late summer community included members of clades CB5, I, IV, and VI. The greater diversity of oligotypes in the late summer relative to early summer is consistent with the late summer communities that occur offshore; at the MVCO site, oligotypes comprising the late summer community were from clades other than clade I, but could not be completely resolved (included matches to CB5, CRD1, II, III, IV, VII, and WPC2; Hunter-Cevera, 2014).

**Figure 7:**
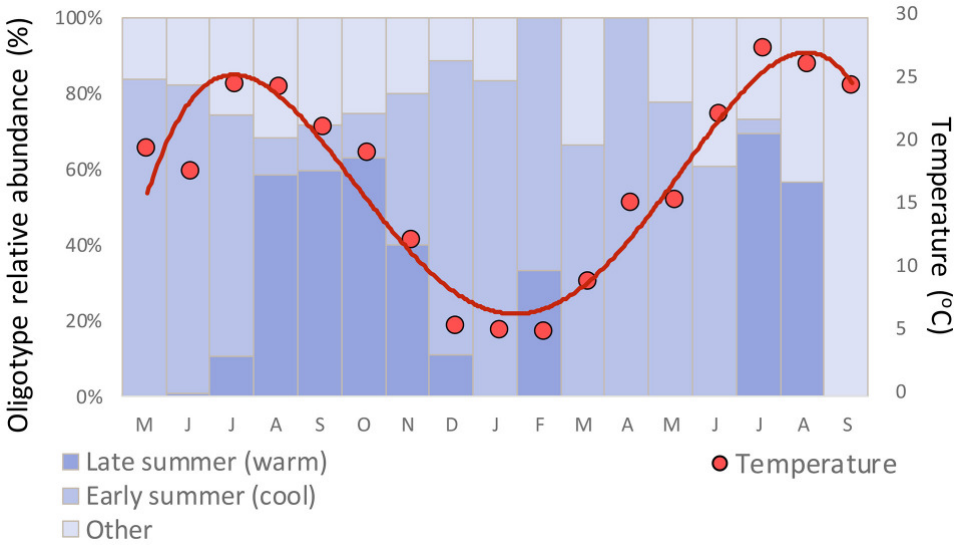
Relationship between seasonal temperature and relative abundance of co-occurring early summer and late summer *Synechococcus* oligotypes.

Clade CB5 that dominated in the late summer in Little Sippewisset Marsh is an estuarine clade that was first identified and described in samples from the Chesapeake Bay (Chen et al., 2006). The observation that this clade emerged strongly under warmer late summer conditions but was not prominent in the cooler early summer period could indicate a preference for warmer water temperatures. While the late summer community shift is correlated with warming temperatures overall (Figure 6), this shift in diversity nevertheless lagged the seasonal warming in that the increase in relative abundance was most pronounced only after temperatures exceeded ~15°C (Figure 7). Similar patterns were observed in LSM for *Pelagibacter*, where the population transitioned from being dominated by a polar oligotype to a tropical oligotype between June and July, and this transition also lagged the seasonal temperature increase (Eren et al., 2013). Other factors not considered in this study, such as nutrient availability, irradiance, viral lysis, and grazing could also influence the observed seasonal dynamics.

The influence of water temperature on dissolved oxygen levels likely explains their anti-correlation (with respect to PC1), and hence greater retention of photosynthetically generated oxygen in the cooler early summer waters compared to warmer late summer temperatures.

### Spatial diversity patterns

Coastal marine waters strongly influenced the physical, chemical, and biological characteristics of Little Sippewissett Marsh waters. The salinity and temperature patterns within the main channel of the estuary closely reflected the seawater end member, as the ocean source is larger than the freshwater source in this estuary. The cyanobacterial community composition along the main channel transect likewise reflected that of the marine source water. This pattern suggests that cyanobacterial populations within the main channel of the estuary were transported from the coastal waters, rather than from the freshwater source, and were either diluted, grazed, or declined due to stress within the estuary.

Greater spatial diversity in *Synechococcus* community structure has been observed in the Chesapeake Bay (Chen et al., 2006) and Pearl River Estuaries (Xia et al., 2015), where communities in coastal and estuarine waters differ considerably. The relatively smaller size of Little Sippewissett Marsh compared to these larger estuaries is likely the reason that *Synechococcus* population structure is more conserved throughout this estuary. While certain characteristics of the *Synechococcus* community are similar to other estuaries, such as having higher diversity and relative abundance in the summer (Xia et al., 2015), spatial differentiation within an estuary may be more sensitive to site characteristics (channel length, residence time, site geomorphology, etc.).

The influence of coastal seawater on the marsh was less pronounced at Station 6, the shallow water station that was more isolated from the main channel. This station supported a diverse population of oligotypes that did not resemble the community composition at the other sites (Figure 5). Certain oligotypes that were dominant in the seawater, such as oligotypes O1 and O2, were also present at station 6. However, their relative abundances were smaller compared to O9 on many of the sampling dates. Oligotype O9 is a member of clade 5.2, which are halotolerant *Synechococcus* more distantly related to marine *Synechococcus* that are commonly found in estuaries and coastal environments (Scanlan et al., 2009). The salinity and dissolved oxygen levels at station 6 were generally similar to other stations within the main channel, although temperature was up to 10°C warmer (Figure 2). Variance in the relative abundance of O9 was not explained by temperature (PC1, Figure 6), despite the fact that higher temperature was a salient feature of the station. This suggests that other factors were more important is driving the relative abundance of O9.

Within the estuary, the community composition at a given site is governed by the net growth rate of each population and the residence time of the water, which determines how quickly populations are diluted by incoming seawater. Limited tidal influence may have enabled the population at station 6 to change relative to the main channel of the estuary by allowing growth to outpace dilution. Less frequent mixing with seawater may have provided sufficient time for the community at station 6 to diverge. Greater exposure to high irradiances, including UV light, in this shallow site would also serve to reshape the microbial community relative to other locations in the estuary and coastal ocean where mixing would mitigate exposure to high light.

These divergent populations could act as reservoirs of diversity for the nearby coastal ocean, particularly following rain events or king tides that would inundate more spatially isolated sites and wash members of the community into the coastal ocean. Indeed, certain rare *Synechococcus* strains have been brought into culture from the nearby MVCO, including phycocyanin-containing strains that lack phycoerythrin (such as sub-cluster 5.2; Hunter-Cevera, 2014). These strains are not abundant at MVCO based on flow cytometry measurements, yet they clearly persist in low numbers in coastal waters and thrive when brought into culture (Hunter-Cevera et al., 2016). Rare microbial populations have the potential to become dominant following local or global changes that favor their growth, hence although they represent only a small fraction of the overall community, their relative abundances could increase in time (Sogin et al., 2006). Spatially isolated sites within local estuaries, like station 6 from within LSM, may be a reservoir of these rare isolates for nearby coastal waters.

Spatial isolation of a site that is more remote from the tidal influence of seawater may also be an important first step in recruiting and enriching unique cyanobacterial communities within the estuary. Prior studies that have characterized microbial communities in LSM have shown that cyanobacteria other than *Synechococcus* (e.g., *Lyngbya, Nostoc, Phormidium*, and *Oscillatoria*) dominate the top layer of microbial mats (Nicholson et al., 1987). Microbial mats are complex communities where co-occurring populations have a high degree of biogeochemical interactivity. Areas like station 6, where unique communities emerge, may therefore also serve as important reservoirs for cyanobacterial diversity in the estuary.

### Using oligotyping to understand interactions among co-occurring *Synechococcus*

An important next step in understanding marine microbial community dynamics is to identify factors that drive the co-occurrence of closely related microbes within an environment, leading certain taxa to co-vary in space and time (Horner-Devine et al., 2007; Fuhrman, 2009). While this is already being done for *Synechococcus* at the sub-clade (Paerl et al., 2011; Robidart et al., 2012) and clade level (Choi and Noh, 2009; Tai and Palenik, 2009; Ahlgren and Rocap, 2012; Sohm et al., 2015), oligotyping allows diversity and succession patterns within microbial communities to be investigated with fine-scale resolution at the nucleotide level. This resolution makes it possible to investigate the underlying dynamics between *Synechococcus* oligotypes that could potentially underpin clade co-occurrence patterns observed in the environment (e.g., clades I and IV) by showing whether oligotypes within the same clade share similar population dynamics.

Our analysis revealed that oligotypes belonging to the same clade often showed similar relative abundance patterns, along with oligotypes from different clades. However, for early and late summer communities, the strength of correlation between oligotypes of the same or different clades varied. For the early summer community, the three most abundant oligotypes (O2, O4, and O5) followed a predictable pattern in which the relative abundances of each oligotype were more or less constant over time (Table S1, Figure S2). This stable proportionality occurred irrespective of whether the oligotypes were from the same clade (as for clade I oligotypes O2 and O4) or different clades (clade IV member O5).

In contrast, the late summer community showed more complex patterns of co-occurrence among oligotypes. The correlations between oligotypes belonging to the same clade were not always strong, and correlations between oligotypes in the CB5 clade from the late summer community provide a good example of this (Table S1; Figure S2). Many studies have shown that the relative proportions of co-occurring *Synechococcus* populations to each other at the clade and subclade level vary in space and time based on environmental factors like seasonal temperature fluctuations (Choi and Noh, 2009; Tai and Palenik, 2009; Xia et al., 2015; Hunter-Cevera et al., 2016), nutrient availability and upwelling (Choi and Noh, 2009; Robidart et al., 2012), circulation patterns (Choi and Noh, 2009; Paerl et al., 2011), and abundance of other phytoplankton (Robidart et al., 2012). This study shows that co-occurrence patterns are also evident at the oligotype level in Little Sippewissett Marsh, and that they change throughout the summer. Greater variability in oligotype co-occurrence behavior was observed in the late summer community compared to the early summer community. This could be due in part to the greater number and diversity of oligotypes comprising the late summer community compared to the early summer community, or the comparatively lower number of sequences for some oligotypes, which complicates correlation analysis.

Rare taxa are recognized as potential reservoirs of genetic diversity (Sogin et al., 2006), and episodic blooms of rare taxa can lead to significant shifts in the biogeochemical and ecological characteristics of the environment (Gilbert et al., 2012). Relative to the more abundant taxa within a microbial community, rare taxa can be closely related (e.g., belonging to the same population or pan-genome) or distantly related (e.g., belonging to different phyla). In both cases rare taxa can confer genetic diversity, but by different mechanisms. Distantly related rare tax may increase genetic diversity in the abundant populations by contributing to their genomes via lateral gene transfer. In contrast, the genomes of rare taxa may differ from closely related abundant taxa by virtue of gene mutation or horizontal gene transfer, which may enable rare taxa to outcompete abundant taxa when environmental conditions shift. Although microbial microdiversity is essential for maintaining proper ecosystem function, our understanding of the environmental factors that drive spatial and temporal changes in rare taxa is still in the early stages (Alonso-Sáez et al., 2015). The oligotyping approach applied here revealed that the late summer *Synechococcus* community included the four least abundant oligotypes, O6, O8, O10, and O11 (Figure S1). The factor that appears to set these rare oligotypes apart from other members of the late summer community is their correlation with higher salinity (PC2; Figure 6), and this was reflected in their distributions within the estuary, where they were never identified in samples from the freshwater source station 4 (Figure S1). It is not clear what factors served to constrain the relative abundances of these rare oligotypes at other sites with higher salinities, but it could stem from nutrient limitation, grazing, or viral susceptibility.

The rare members of the community did not follow any consistent pattern in terms of their correlations with each other or the more dominant oligotypes. Of the four oligotypes, O10 was generally the least correlated with other oligotypes in the late summer community (Table S1, Figure S2) despite being a member of clade CB5. Hence, despite being closely related at the clade level to many other co-occurring oligotypes in the community, this rare oligotype displayed different relative abundance patterns. This microdiversity may effectively permit clade CB5 to fill a slightly larger niche than if it contained fewer oligotype members.

We observe that the relative abundance of co-occurring *Synechococcus* is not constant over time; the timing with which each oligotype reaches its peak relative abundance during the late summer is slightly offset. In the late summer community, oligotype O7 shows this pattern, where its correlation with oligotypes O8, O10, and O11 shows a bifurcated pattern (Figure S2) that is driven by O7 reaching its peak relative abundance earlier in the season than the other oligotypes.

This study investigated the seasonal community dynamics of *Synechococcus* oligotypes in a small New England estuary. Patterns that have been observed at the clade and subclade levels, such as correlation between temperature and *Synechococcus* relative abundance, and the co-occurrence of groups from different clades, were shown to occur among oligotypes. This analysis still leaves open the question of whether co-occurring oligotypes affect each other in beneficial ways, as is common in microbial mats within Little Sippewissett Marsh. The phenomenon of co-occurrence appears to be a case of “Paradox of the Plankton (Hutchinson, 1961);” how are very similar organisms able to coexist simultaneously? *Synechococcus* microdiversity is an excellent case study of such microbial diversity questions, and how such diversity is maintained. Do clades differ slightly in physiology, or do ecological selection factors make this co-occurrence possible? While at times oligotypes within the same and different clades do co-exist, their relative abundances change over the season. As hypothesized by Hutchinson (1961), changes in the environment (such as the spring warming) appear to prevent any one clade from excluding all others. Further application of *Synechococcus* oligotyping in other environments may shed light on these long standing questions by providing new insights on (1) the potential competitive, commensal, and mutualistic interactions that may be occurring among closely related *Synechococcus* oligotypes, (2) the role of rare oligotypes within the community, and (3) the drivers of *Synechococcus* biogeography under current and future changing environmental conditions.

## Author contributions

All authors listed have made substantial, direct, and intellectual contribution to the work and approved it for publication.

### Conflict of interest statement

The authors declare that the research was conducted in the absence of any commercial or financial relationships that could be construed as a potential conflict of interest.
