# Supplementary material for: Seasonal Succession and Spatial Patterns of Synechococcus Microdiversity in a Salt Marsh Estuary Revealed through 16S rRNA Gene Oligotyping
Source: Front Microbiol. 2017 Aug 9;8:1496. doi: 10.3389/fmicb.2017.01496 (PMC5552706; doi:10.3389/fmicb.2017.01496)

Figure S1: Monthly relative abundance of each *Synechococcus* oligotype (relative to total *Synechococcus* counts for each sample). Note different scales are used for rare oligotypes O6, O8, O10, and O11.

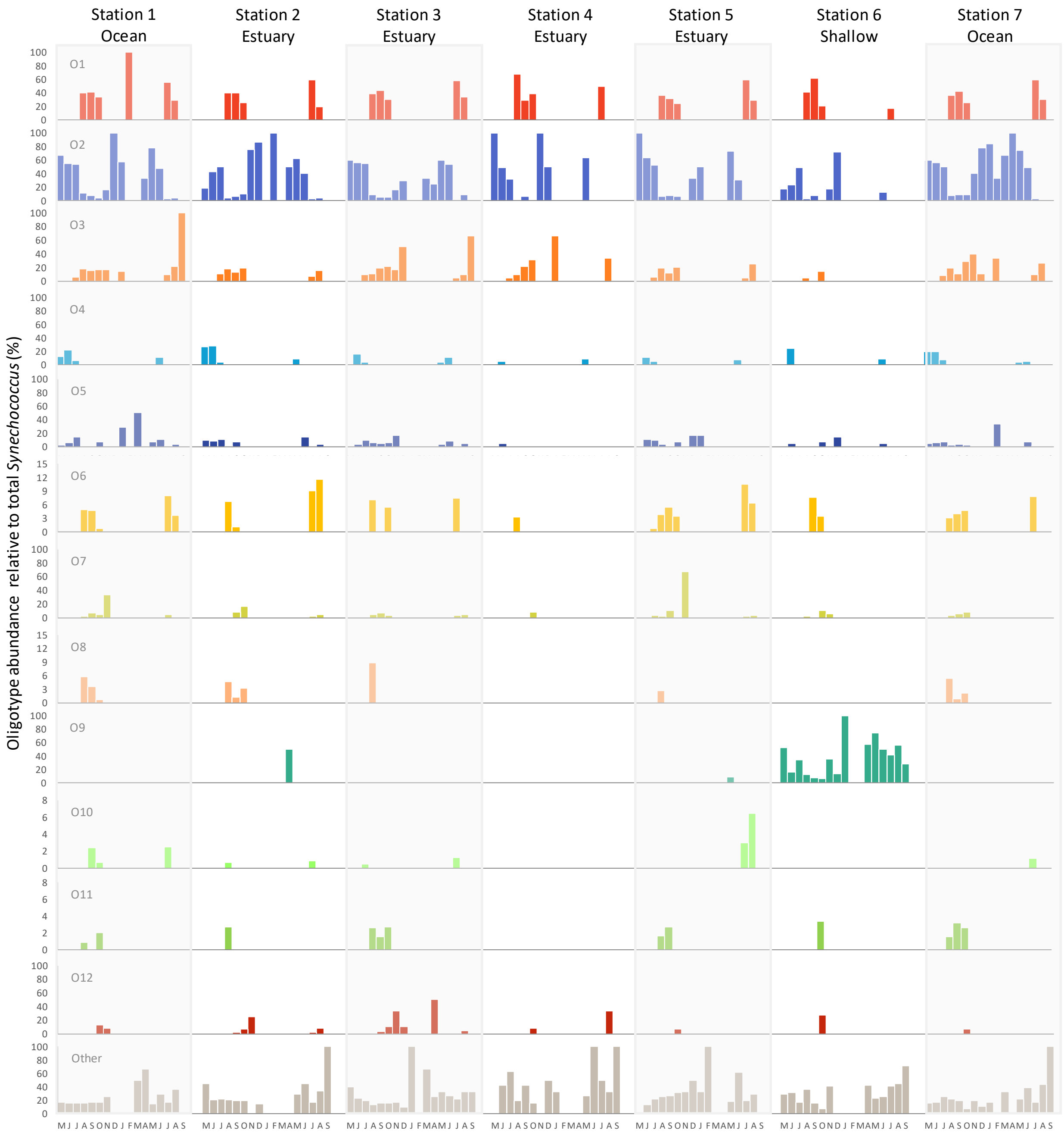

Supplement: Supplementary file 1 [file Image1.pdf]
