# Supplementary material for: Seasonal Succession and Spatial Patterns of Synechococcus Microdiversity in a Salt Marsh Estuary Revealed through 16S rRNA Gene Oligotyping
Source: Front Microbiol. 2017 Aug 9;8:1496. doi: 10.3389/fmicb.2017.01496 (PMC5552706; doi:10.3389/fmicb.2017.01496)
Supplement: Supplementary file 2 [file Image2.pdf]

Figure S2: Correlations among *Synechococcus* oligotypes. Co-occurring early summer oligotypes are shown in blue, and co-occurring late summer oligotypes are shown in red. All other pairs are shown in grey. (Figure is split between two pages.)

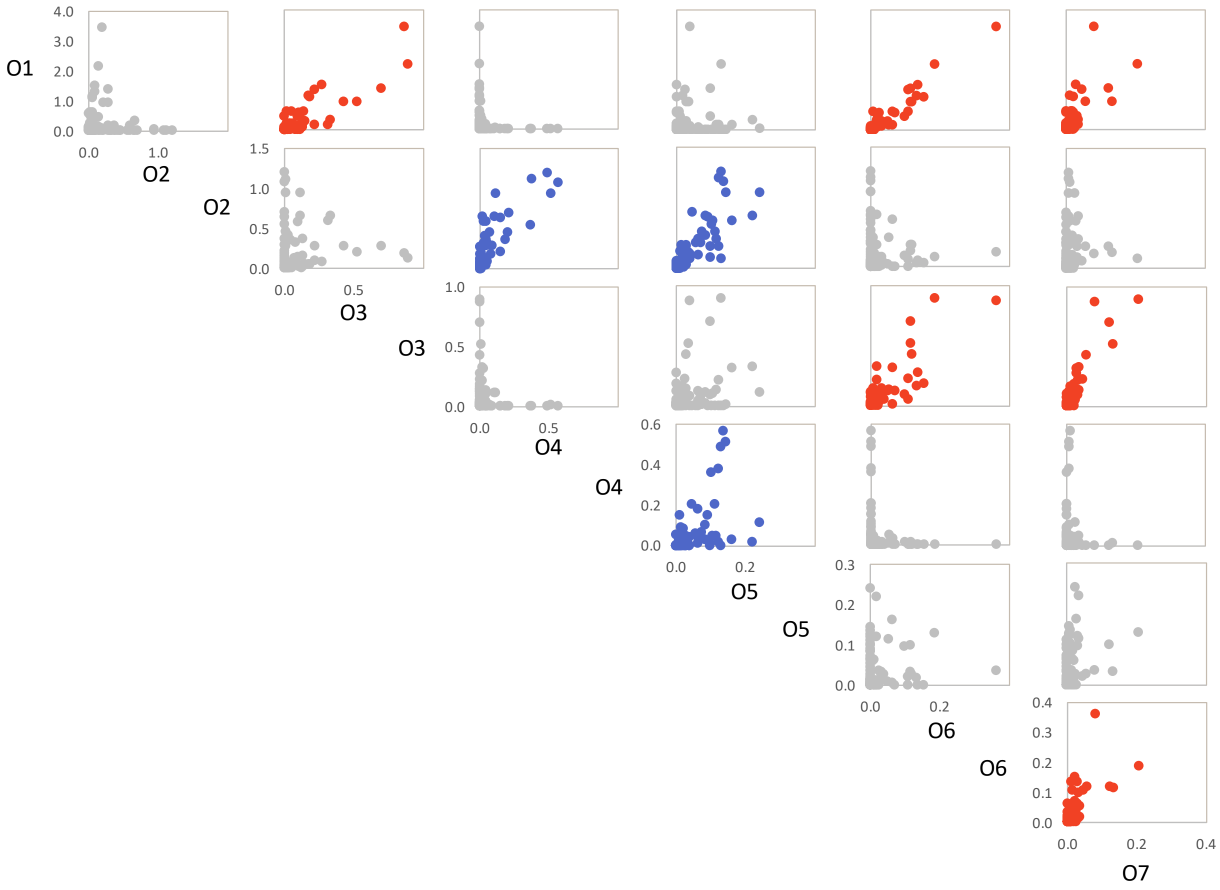

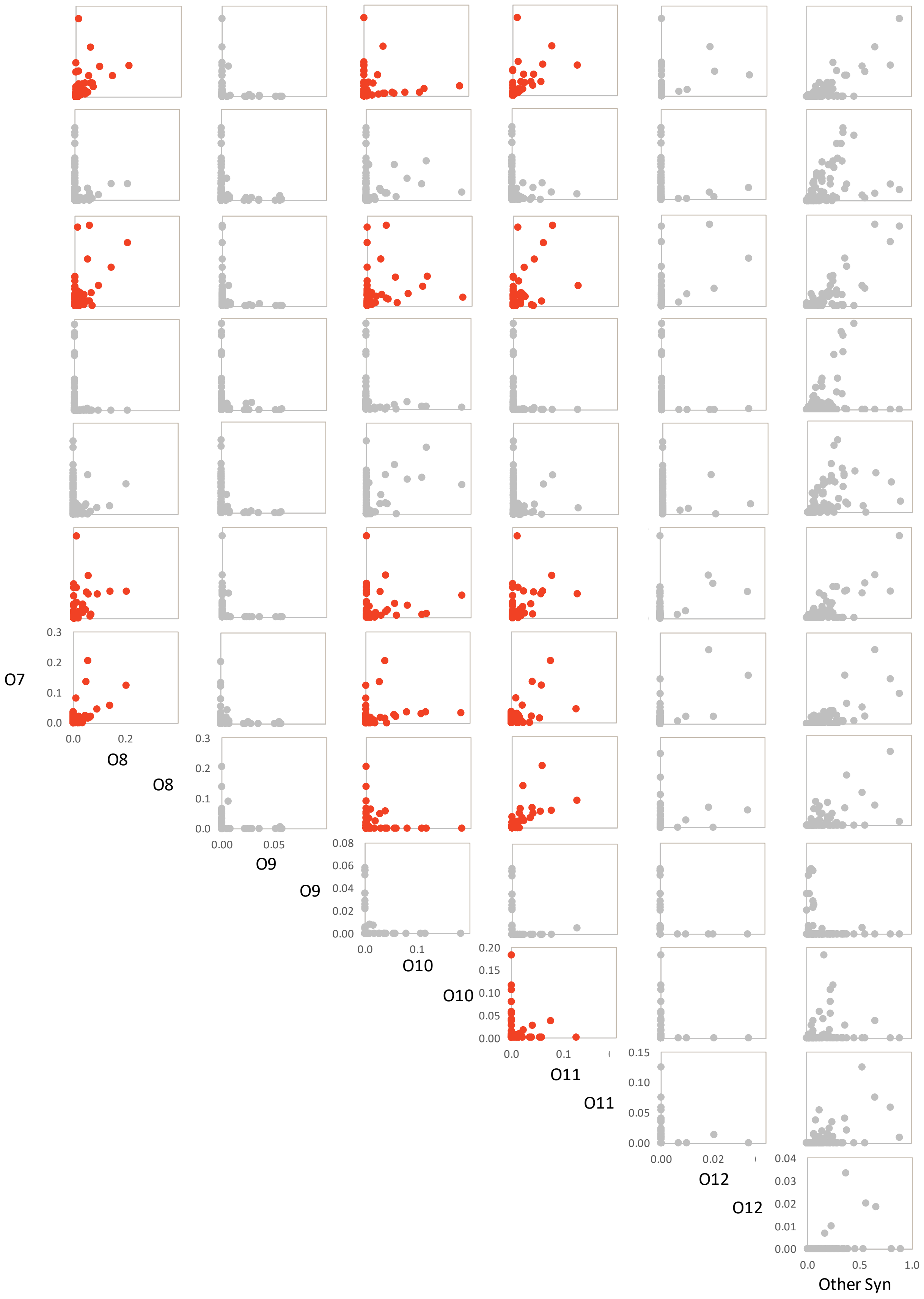
O7
0.3
0.2
0.1
0.0
0.0
0.2
O8
O8
0.3
0.2
0.1
0.0
0.00
0.05
O9
O9
0.08
0.06
0.04
0.02
0.00
0.0
0.1
O10
O10
0.20
0.15
0.10
0.05
0.00
0.0
0.1
O11
O11
0.15
0.10
0.05
0.00
0.00
0.02
O12
O12
0.04
0.03
0.02
0.01
0.00
0.0
0.5
1.0
Other Syn
