# Supplementary material for: Seasonal Succession and Spatial Patterns of Synechococcus Microdiversity in a Salt Marsh Estuary Revealed through 16S rRNA Gene Oligotyping
Source: Front Microbiol. 2017 Aug 9;8:1496. doi: 10.3389/fmicb.2017.01496 (PMC5552706; doi:10.3389/fmicb.2017.01496)
Supplement: Supplementary file 3 [file Table1.pdf]

Table S1: Correlations between *Synechococcus* oligotype relative abundance (relative to total microbial reads). Co-occurring early summer oligotypes are in blue, and co-occurring late summer oligotypes are in red.

	**1**	**2**	**3**	**4**	**5**	**6**	**7**	**8**	**9**	**10**	**11**	**12**
**1**		0.11	.87	0.19	.05	.96	.72	.48	0.15	.08	.56	.36
**2**			.06	.83	.83	0.09	.07	0.08	0.21	.09	0.12	0.06
**3**				0.16	.31	.83	.91	.55	0.15	.24	.51	.46
**4**					.53	0.19	0.11	0.16	0.13	0.10	0.15	0.09
**5**						.06	.32	.02	0.23	.42	.01	.03
**6**							.68	.41	0.16	.16	.47	.37
**7**								.58	0.13	.27	.60	.62
**8**									0.11	0.06	.71	.14
**9**										0.11	0.02	.30
**10**											0.02	.07
**11**												.30
**12**												
